# Intrahepatic cholangiocarcinoma treated with glass yttrium-90 radioembolization: A histopathologic study

**DOI:** 10.1007/s00259-026-07996-y

**Published:** 2026-07-02

**Authors:** Qian Yu, Peizi Li, Satvik Jain, Abdul Khan, Joseph Steiner, Chih-Yi Liao, Joseph Franses, Anjana Pillai, John Fung, J Michael Millis, Rakesh Navuluri, Steven Zangan, Brian Funaki, John Hart, Osman Ahmed, Thuong Van Ha

**Affiliations:** 1 Department of Radiology, University of Chicago Medical Center, University of Chicago, Chicago, IL 60637 USA; 2 Department of Pathology, University of Chicago Medical Center, University of Chicago, Chicago, IL 60637 USA; 3 School of Medicine, University of Chicago, Chicago, IL 60637 USA; 4 Department of Medicine; Hematology and Oncology, University of Chicago Medical Center, University of Chicago, Chicago, IL 60637 USA; 5 Division of Gastroenterology, Hepatology, and Nutrition, University of Chicago Medical Center, University of Chicago, Chicago, IL 60637 USA; 6 Department of Surgery, The University of Chicago Medicine, Chicago, IL 60637 USA

**Keywords:** Cholangiocarcinoma, Intrahepatic, Pathology, Radioembolization, Yttrium-90

## Abstract

**Purpose:**

To determine the degree of tumor necrosis on pathology and clinical outcomes among intrahepatic cholangiocarcinoma (iCCA) patients who were treated with transarterial radioembolization (TARE) and the bridge or downstaged to surgery.

**Methods:**

A single center retrospective review was performed to include consecutive patients who underwent TARE using glass microspheres and then subsequently underwent surgical resection or transplant. Baseline characteristics, dosimetry, radiologic response, histological tumor necrosis, and overall survival were evaluated. A total of 20 patients (F: M = 14:6; age: 57.3 ± 10.8 years) underwent TARE and then received resection (*n* = 15) or living-donor liver transplant (*n* = 5). A total of 23 selective TARE (two segments or less) were delivered, with a mean dose 301.7 ± 177.5, whereas 18 lobar (left or right hepatic artery) doses were delivered, with a mean dose of 150.3 ± 44.1 Gy. Concurrent systemic therapy was administered in 15 patients.

**Results:**

The mean time from the initial TARE to surgery was 7.1 ± 9.2 months. On pathology, complete tumor necrosis, > 90% necrosis, and extensive tumor necrosis (> 50%) were achieved in 1 (5.0%), 13 (65.0%), and 18 (90.0%) patients, respectively. The median survival time (MST) calculated from the time of surgery was 40.2 (95%CI: 15.3-not reached) months, with a 1-yr, 3-yr, and 5-yr OS of 88.2% (95CI: 60.6–96.9%), 57.8% (95%CI: 31.1–77.3%), and 19.8% (95%CI: 1.5–53.5%). From the time of initial TARE, the MST was 41.4 (95%CI:22.9-not reached) month, with a 1-yr, 3-yr, and 5-yr OS of 100%, 69.9% (95%CI: 42.0-86.3%), and 30.9% (95%CI: 9.1–56.3%). Whether the patient received concomitant systemic treatment was associated with extensive tumor necrosis on pathology (Odds Ratio: 16.0, 95%CI: 1.27-200.92, *p* = 0.032).

**Conclusion:**

Based on tumor explant data, TARE can result in substantial tumor necrosis in the treatment of iCCA, although complete necrosis is uncommon. The addition of systemic therapy appears to be associated with a higher likelihood of achieving extensive tumor necrosis.

## Introduction

The incidence of intrahepatic cholangiocarcinoma (iCCA) in the United States has tripled over the past two decades. However, prognosis remains poor, with 5-year overall survival (OS) rates of 28.2%, 12.1%, and 3.0% for patients with localized, regional, and distant disease, respectively [1]. For patients with advanced, liver-limited iCCA that is initially unresectable, transarterial radioembolization (TARE) has been shown to be effective in achieving tumor control and in serving as a bridge or downstaging strategy to surgery [2–7]. Ablative, radiation segmentectomy > 400 Gy and radiation lobectomy > 120 Gy have demonstrated both safety and efficacy in iCCA [3, 8]. According to international consensus guidelines, a single-compartment average perfused volume absorbed dose ≥ 400 Gy is recommended for radiation segmentectomy and ≥ 150 Gy for radiation lobectomy [9].

While prior studies have reported on the radiologic response and survival outcomes associated with TARE in the treatment of iCCA [4, 8, 10–13], data on the degree of pathologic tumor necrosis following TARE remain limited. This information is important, as understanding the relationship between radiation dose and pathologic response may help guide the development of standardized dosimetry strategies for iCCA. According to a recently published retrospective single-center study of 18 patients with iCCA treated with TARE using resin microspheres, complete pathologic necrosis was achieved in 22% of patients and extensive necrosis (> 90%) in 72%, with higher necrosis rates observed in patients receiving a prescribed dose ≥ 180 Gy and in those treated with first-line TARE [14]. However, histopathologic data for glass microspheres remain scarce. The aim of the present study was to evaluate the extent of pathologic tumor necrosis in patients with iCCA who were treated with TARE using glass microspheres and subsequently underwent surgical resection or liver transplantation.

## Methods

This study was performed in line with the principles of the Declaration of Helsinki. Approval was granted by the Ethics Committee of University of Chicago. This is a retrospective, observational study, and therefore written consent was waived per IRB.

### Selection criteria

A single center retrospective review was performed to include consecutive patients who underwent transarterial radioembolization using glass microspheres from December 2018 to August 2025, and then subsequently underwent surgical resection or transplant.

### Technique

Radioembolization was performed in two outpatient encounters consisting of a mapping phase and a treatment delivery phase, as previously described [10, 15]. All TARE procedures were performed using glass microspheres (TheraSphere, Boston Scientific) with dosimetry calculated using the Medical Internal Radiation Dose model. Over time, the prescribed dose increased in response to emerging literature supporting the safety and effectiveness of ablative radiation segmentectomy (> 400 Gy) and radiation lobectomy (> 80–120 Gy) in 2021 [16, 17].

The treatment goals and approach for each case were discussed at a weekly multidisciplinary conference that included specialists in hepatology, medical oncology, transplant surgery, hepatobiliary surgery, diagnostic radiology, and interventional radiology.

In general, treatment-naïve patients with locally advanced iCCA received first-line combination therapy with gemcitabine, cisplatin, capecitabine, and TARE, as previously described [10]. Following publication of the TOPAZ-1 trial, durvalumab was added to the treatment regimen [18]. For patients who initially underwent systemic therapy at outside hospitals and were subsequently referred to the authors’ institution, second-line systemic therapy was combined with TARE in cases of progressive tumor burden, provided the patient was able to tolerate further systemic treatment. Systemic therapy was generally held for 3–4 weeks prior to TARE. Patients were evaluated for surgical resection if tumor burden decreased on follow-up imaging. In patients whose disease remained confined to the liver after 6–9 months, living-donor liver transplantation could be considered.

### Pathologic evaluation techniques for liver explant specimens

For macroscopic evaluation, all explanted livers are received fresh and are dissected into 5 mm slices. All slices are reviewed grossly and thoroughly to look for abnormalities such as residual solid tumors, hemorrhagic areas, necrotic areas, or Y-90 beads, etc. The background liver is also assessed for incidental nodules or fibrosis. All residual liver tumors are identified and measured in three dimensions, and grossly hemorrhagic dark areas are also measured and assessed for tumor necrosis.

All identified tumors are representatively sectioned to show the relationship with the liver capsule, liver hilar areas, or adjacent liver tissue. Additional sections are performed as needed to ensure that any areas of potentially viable tissue are evaluated. All sections are fixed in formalin, embedded in paraffin, and undergo microscopic evaluation. Based upon the combination of macroscopic 5 mm sections, selected microscopic 3 μm sections, and additional sections of potentially viable tissue throughout the tumor, post-treatment tumor necrosis is determined visually to the nearest 5% (i.e., 0%, 5%, …, 95%, 100%) and later further subclassified into complete, extensive, and partial pathologic tumor necrosis, defined as 100%, > 90%, and > 50% tumor necrosis, respectively. If a lesion does not initially demonstrate any evidence of viable tumor by these means, the entire tumor is submitted for 3 μm sections to fully assess tumor necrosis.

**Fig. 1:**
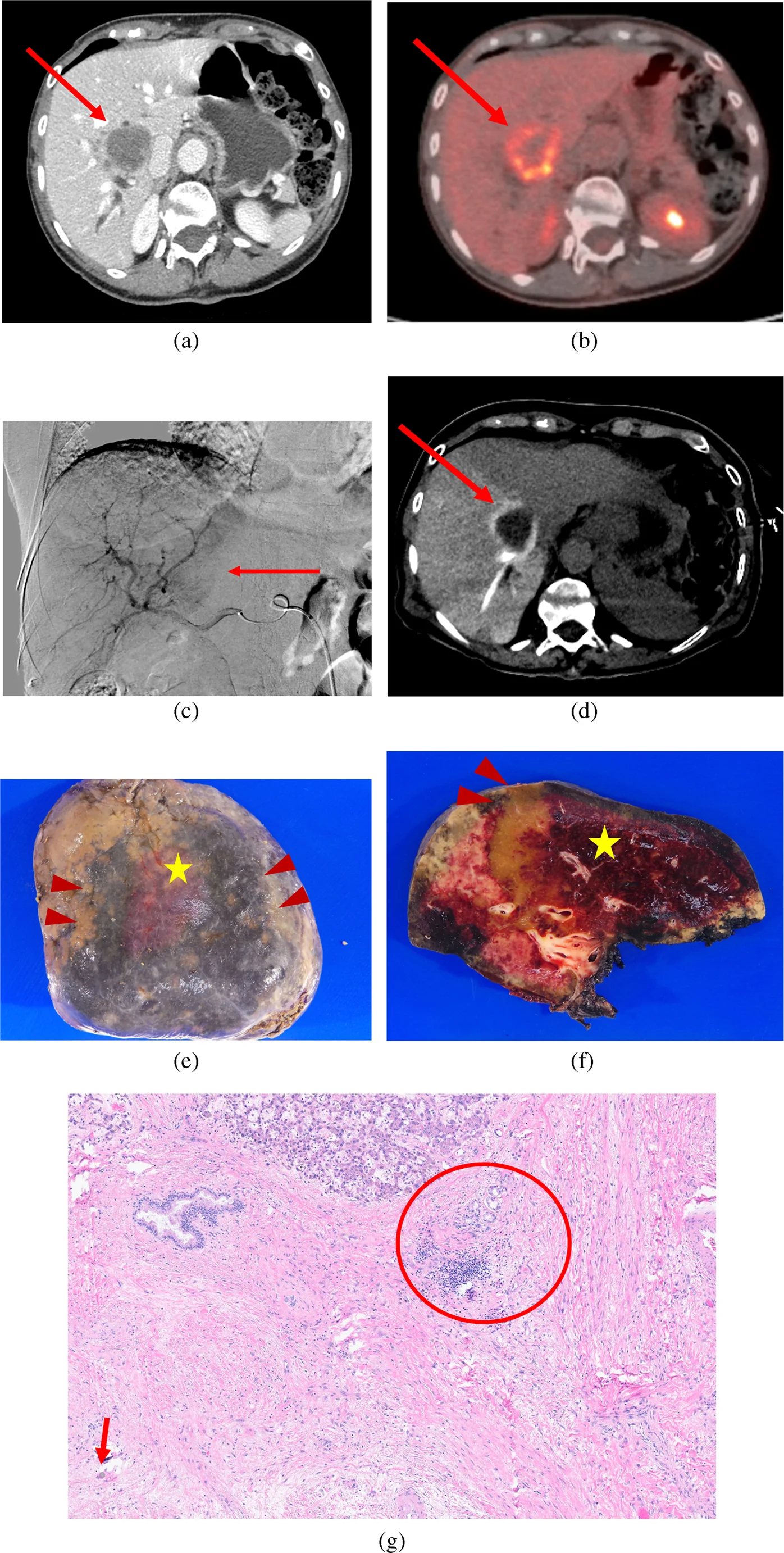
(**a**) Computed tomography showed a biopsy-proven intrahepatic cholangiocarcinoma (red arrow)located near the inferior vena cava. (**b**) The tumor (red arrow) was FDG avid on PET. (**c**) catheter angiogram showed tumor vascularity (red arrow) supplied by the right hepatic artery. (**d**) Intraprocedural Angio-CT showed y90 delivery coverage of the tumor (red arrow). (**e**, **f**) gross specimen of extended right hepatectomy. The liver capsule is intact. There is a distinct necrotic area (red arrow heads) measuring 8 × 8 × 3 cm, with a central hemorrhagic area (yellow star) within the necrotic region measuring 5 × 3 cm. (**g**) Microscopy showed Y90 beads (red arrow) embedded within the necrotic area and microscopic foci of residual viable cholangiocarcinoma (red circle) in the background of extensive necrosis and fibrosis

**Fig. 2:**
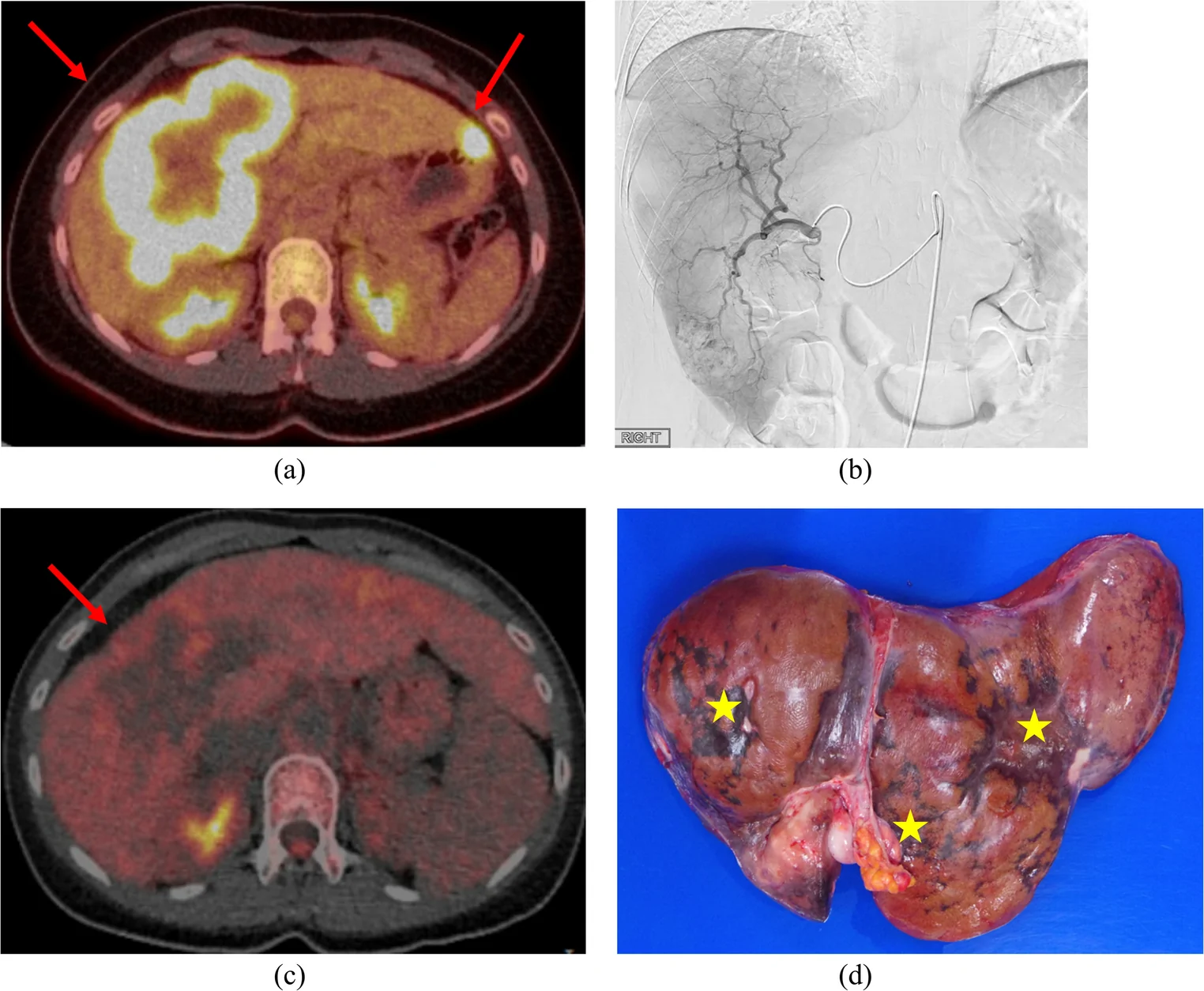
(**a**) PET/CT showed bilobar FDG-avid multifocal intrahepatic cholangiocarcinoma (red arrow). (**b**) This patient received sequential right and left radiation lobectomy. (**c**) Post-treatment PET/CT showed significantly decreased FDG avidity (red arrow). (**d**) This patient underwent living-donor liver transplant. Tumor explant showed multifocal treatment effect (yellow star) on the liver capsule

Examples were shown in Figs. 1 and 2.

### Variables and outcomes

Baseline characteristics including patient age, sex, disease burden, radiation dose, radiologic response, surgery type were collected. The best radiologic response was evaluated per RECIST 1.1 and PERCIST criteria. In general, radiologic response was evaluated with contrast enhanced computed tomography or magnetic resonance imaging 6–8 weeks after TARE, and every 3 months afterwards.

The primary outcome of interest was histological tumor necrosis. Complete, extensive, and partial pathologic tumor necrosis was defined as 100%, > 90%, and > 50% tumor necrosis. Secondary outcomes include overall survival (OS), calculated from the time of TARE and surgery, respectively, surgical complications, and post-surgical recurrence.

### Statistical analysis

Continuous variables were reported as mean ± standard deviation, and categorical variables were reported as counts and percentages. Baseline variables between patients with and without extensive pathologic tumor necrosis were compared using the chi-square test or Fisher’s exact test for categorical variables and the t-test for continuous variables. Logistic regression analysis was performed to identify variables associated with extensive pathologic tumor necrosis. OS was analyzed using the Kaplan–Meier method and reported as median OS as well as OS at 1, 3, and 5 years. Statistical significance was defined as a p-value < 0.05. All statistical analyses were performed using STATA/MP 18 (StataCorp, College Station, TX).

## Results

### Demographics

A total of 20 patients were included in the present study (14 females, 6 males) with a mean age of 57.3 ± 10.8 years (Table 1). Multifocal disease was present in 5/20 (25.0%) patients; 12/19 tumors were located centrally or had bilobar distribution (63.2%), whereas 1 patient presented with tumor recurrence after prior surgical lobectomy. The mean dominant tumor size was 5.4 ± 2.7 cm.

**Table 1:** Demographics and outcomes

Variables	Value
Sample size	20
Female	14
Male	6
Age (years)	57.3 ± 10.8
Multifocal disease	5/20 (25.0%)
Central or Bilobar Distribution	12/19 (63.2%)
Dominant Tumor Size (cm)	5.4 ± 2.7
Dose (Gy)	
Segmental (n=23)	301.7 ± 177.5
Lobar (n=18)	150.3 ± 44.1 Gy
Type of Surgery	
Right trisegmentectomy	4
Right trisegmentectomy+segment 1 resection	2
Right hepatectomy	5
Extended left trisegmentectomy	2
Left hepatectomy	1
Segmentectomy	1
Living-donor Liver Transplant	5
Tumor Necrosis	
Complete (100%)	1/20 (5%)
Extensive (>90%)	13/20 (65%)
>50%	18 (90%)
Negative Surgical Margin	12/15 (80%)
Time from Y90 to Surgery (months)	7.1 ± 9.2
Post-surgical Follow-up (months)	30.5 ± 20.9

Seven patients received more than one Y90 administration sessions (35%). A total of 23 segmental doses were delivered, with a mean dose of 301.7 ± 177.5 Gy. A total of 18 lobar doses were delivered, with a mean dose of 150.3 ± 44.1 Gy. Concurrent systemic therapy was administered in 15/20 patients (75%).

The following surgeries were performed: Right trisegmentectomy (*n* = 4), right trisegmentectomy+segment 1 resection (*n* = 2), right hepatectomy (*n* = 5), extended left trisegmentectomy (*n* = 2), left hepatectomy (*n* = 1), segmentectomy (*n* = 1), LDLT (*n* = 5).

### Pathologic necrosis

Complete pathologic tumor necrosis was achieved in 1/20 patients (5%). Extensive tumor necrosis was achieved in 13/20 (65%). Eighteen patients had at least 50% tumor necrosis on pathology (90%).

Among tumors with extensive pathologic necrosis, a higher percentage of patients received concomitant systemic treatment (92.3% vs. 42.9%, *p* = 0.031; Table 2). In this group, a greater percentage of tumors showed radiologic response on follow-up (100% vs. 42.9%, *p* = 0.031). On logistic regression analysis, whether the patient received concomitant systemic treatment was associated with extensive tumor necrosis on pathology (OR: 16.0, 95%CI: 1.27-200.92, *p* = 0.032, Table 3).

**Table 2:** Comparison of baseline characteristics among tumors with and without extensive tumor necrosis

Variables	>90% (*n* = 13)	<90% (*n* = 7)	*P* value
Age	57.4 (10.3)	57.1 (12.6)	0.964
Dominant Tumor Size >5cm	8/13 (61.5%)	1/7 (14.3%)	0.07
Bilobar	6/13 (46.2%)	5/6 (83.3%)	0.333
Multifocal	3/13 (23.1%)	2/7 (28.6%)	1.00
Concurrent Systemic Treatment	12/13 (92.3%)	3/7 (42.9%)	0.031
>200Gy	7/11 (53.9%)	5/7 (71.4%)	0.642
Response Rate (RECIST)	13/13 (100%)	4/7 (42.9%)	0.031
TARE to Surgery (days)	275.6 (329.7)	100.6 (19.9)	0.185

**Table 3:** Logistic regression analysis between different variables and whether extensive tumor necrosis was achieved

Variables	OR	95%CI	*P* value
Age	1.00	0.92-1.09	0.961
Tumor Size >5cm	9.6	0.88-105.17	0.064
Bilobar	0.233	0.021-2.59	0.236
Multifocal	0.75	0.093-6.04	0.787
Concurrent Systemic Treatment	16.0	1.27-200.92	0.032
Dose >200Gy	0.467	0.065-3.34	0.448
Response Rate	21	0.90-489.76	0.058
Time from TARE to Surgery	1.02	0.996-1.05	0.098

### Survival

**Fig. 3:**
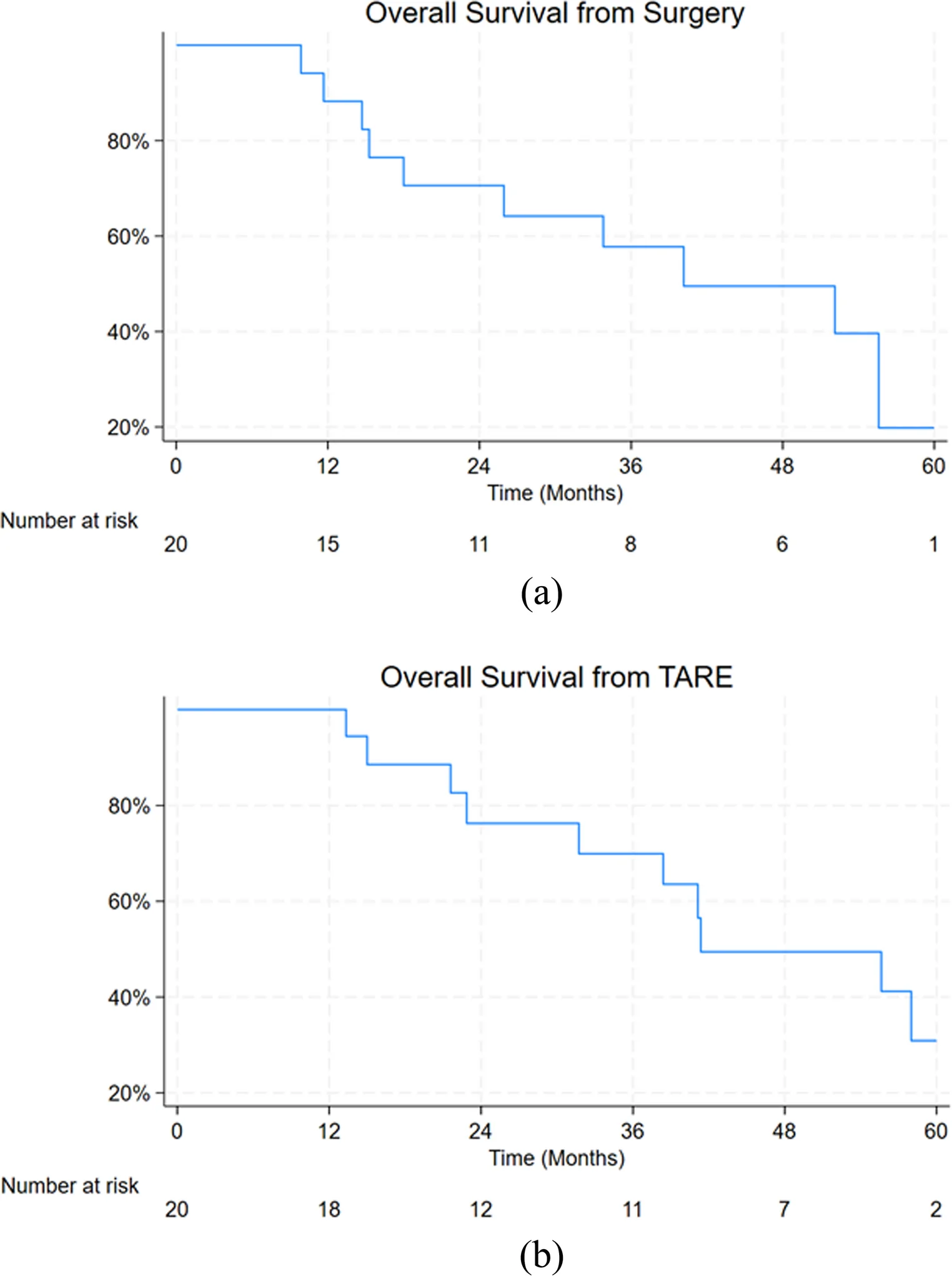
(**a**) Overall survival calculated from the time of surgery. (**b**) Overall survival calculated from the time of radioembolization

Median survival time (MST) from the date of surgery was 40.2 months (95% CI: 15.3–not reached), with 1-, 3-, and 5-year OS rates of 88.2% (95% CI: 60.6–96.9%), 57.8% (95% CI: 31.1–77.3%), and 19.8% (95% CI: 1.5–53.5%), respectively (Fig. 3a).

From the time of initial TARE, MST was 41.4 months (95% CI: 22.9–not reached, Fig. 3b), with 1-, 3-, and 5-year OS rates of 100%, 69.9% (95% CI: 42.0–86.3%), and 30.9% (95% CI: 9.1–56.3%).

**Fig. 4:**
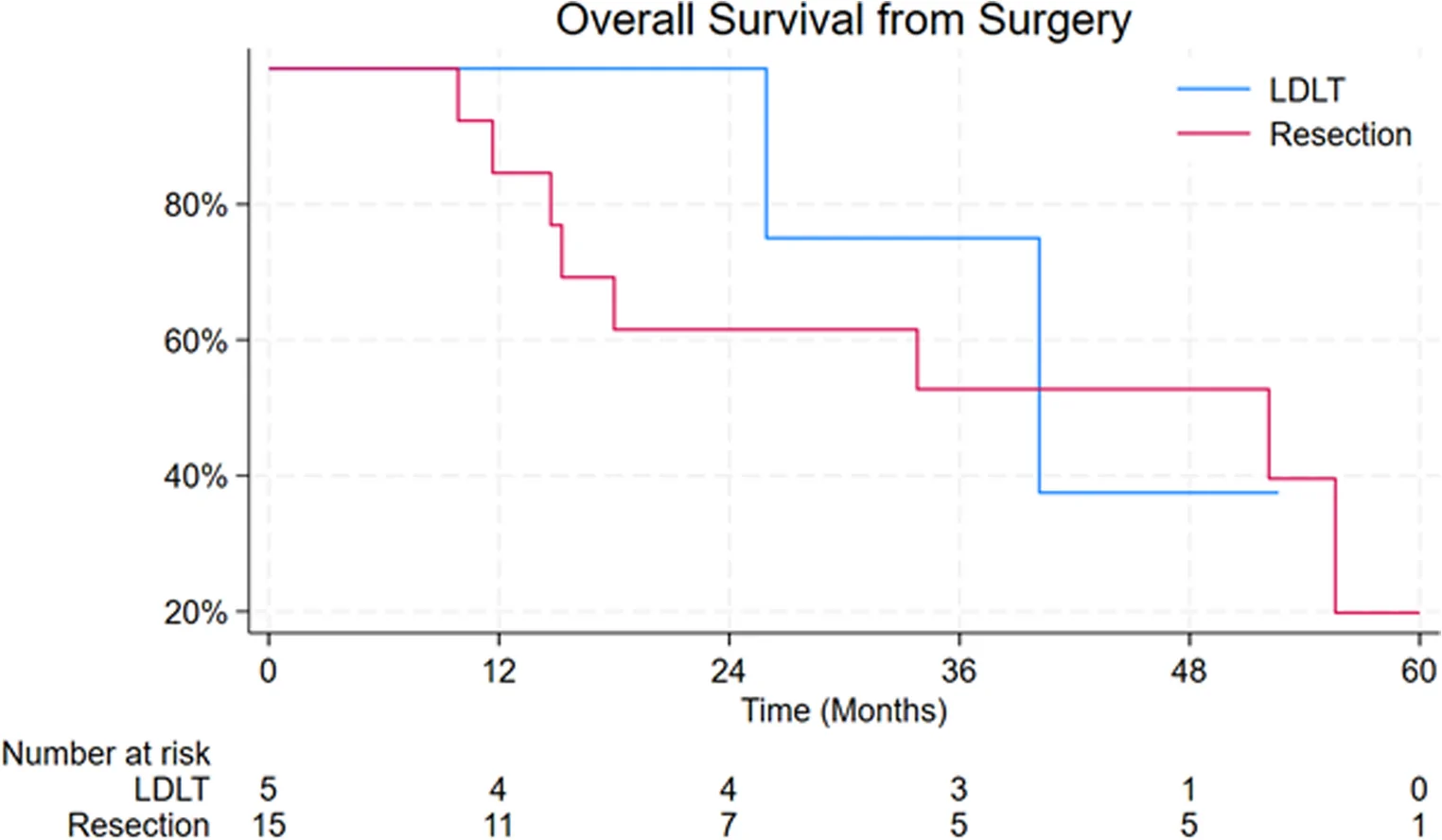
Overall survival of patients who underwent resection and living-donor liver transplant

The median OS among patients who underwent resection was 40.2 (95%CI: 25.3-not reached) months, with a 1- and 3-yr OS of 84.6%(95%CI: 51.2–95.9%) and 52.8%(95%CI: 23.4–75.5%) after resection (Fig. 4). The median OS of the LDLT was 52.1 (95%CI: 14.7-not reached) months, with a 1- and 3-yr OS of 100% and 75.0% (95%CI: 12.8–96.1%) after transplant. No significance difference was noted in two subgroups (Log-rank *p* = 0.711).

### Radiologic response after TARE

Based on RECIST 1.1 criteria, partial response (PR) was achieved in 17/20 patients (85.0%), whereas stable disease (SD) was observed in 3 patients (15.0%), yielding a response rate and disease control rate of 85% and 100%, respectively. Among patients with PR, 13/17 achieved extensive pathologic necrosis (76.5%), whereas none of patients in the SD group achieved extensive pathologic necrosis.

Among 10 patients who underwent post-TARE PET-CT prior to surgery, six (60.0%) showed no residual FDG avidity. Of these, five had > 90% tumor necrosis on pathology, and one had 80% necrosis. Four patients (40.0%) demonstrated residual FDG avidity after TARE; of these, three had > 90% tumor necrosis, one had 50% necrosis. Among 8 patients with both pre- and post-TARE PET-CT available, five (62.5%) achieved a complete response (CR) and three (37.5%) achieved a partial response (PR) according to PERCIST criteria.

### Post-surgical recurrence

Recurrence occurred in 9 patients (60%) after resection. Among 15 patients with resection, R0 resection was achieved in 12 patients (80%). Three patients had positive margin. Two patients developed progressive and extrahepatic disease, despite adjuvant systemic therapy. One patient with microscopic tumor foci at the margin was treated with adjuvant chemoradiation and has been disease free.

Intrahepatic progression occurred in 4 patients, among which 3 recurrences were seen along surgical margins; one had R1 resection, while the other two had R0 resection but the distance of tumor to surgical margin was 1 mm. The other patient had R0, and intrahepatic tumor progression was in the liver remnant away from the resection margin. Among the five patients who developed extrahepatic disease, lymph node dissection was performed in two patients at the time of surgery, which were positive in one case. Resection margin was positive in an additional case.

Three patients developed bile leak after resection (20.0%). One patient developed hepatic decompensation after right trisegmentectomy, requiring orthotopic liver transplant.

**Fig. 5:**
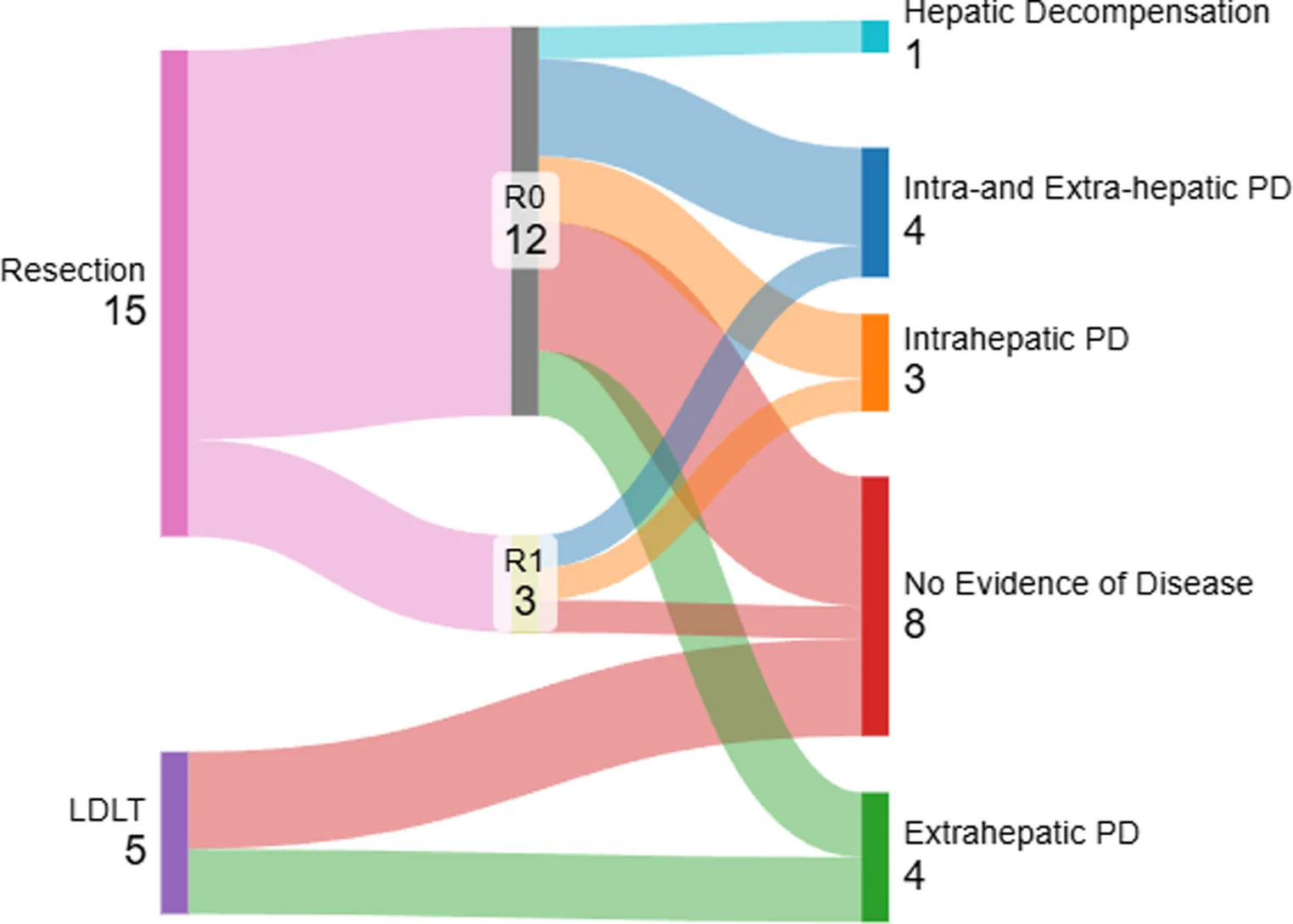
Post-surgical outcomes (Sankey Diagram)

Five patients received LDLT. Two patients developed extrahepatic metastases on follow-up, including metastases in the lung. One was treated with pemigatinib and radiation therapy, whereas the other was treated with gemcitabine/oxaliplatin. Detailed post-surgical outcomes were depicted in Fig. 5.

## Discussion

This histopathologic study demonstrated that TARE using glass microspheres for the treatment of iCCA can achieve a considerably high degree of tumor necrosis, with 65% of cases showing extensive necrosis. Tumors exhibiting a radiologic response and those receiving concurrent systemic therapy were associated with a higher likelihood of extensive tumor necrosis. However, post-surgical recurrence remains common.

The majority of current TARE dosimetry approaches for iCCA have been extrapolated from data in HCC. Given the available histopathologic tumor necrosis data in HCC, dose thresholds for the treatment of iCCA have been adapted according to international expert recommendations [9]. Yet, compared with the literature on HCC, the degree of pathologic necrosis observed in iCCA appears substantially lower [16]. First, iCCA is typically less vascular than HCC, which may limit microsphere deposition. Additionally, the tumor burden in the present study is greater than that reported in many HCC series. Advanced iCCA is often diagnosed at a later stage and frequently has multiple arterial supplies, whereas HCC is more likely to be detected earlier through cirrhosis surveillance. Furthermore, a substantial proportion of tumors in this study had been previously treated with systemic therapy or surgical resection. The response of iCCA to systemic therapy is generally poorer than the response of HCC to TARE [18, 19]. Collectively, these factors may have contributed to the lower rate of tumor necrosis observed in this study.

Because of the heterogeneity in tumor burden and treatment history, a clear dose threshold could not be identified. From a technical standpoint, the iCCA cases included in this study often presented as large, centrally located, bilobar, and multifocal tumors. These lesions frequently have multiple arterial supplies, necessitating more than one treatment delivery. In such scenarios, dose calculation becomes challenging, as different regions of the tumor receive varying radiation doses. By comparison, prior dose–pathology studies in HCC have generally involved smaller tumors, for which a single treatment delivery is often sufficient [16, 20]. Although absorbed dose could, in principle, be used to evaluate dose–pathology relationships in cases with multiple deliveries, this approach typically requires PET/CT imaging, which has not yet become standard of care at the authors’ institution.

Radiologic response is a well-established factor associated with the degree of tumor necrosis on pathology. A single-center retrospective analysis of 33 patients with HCC who underwent Y90 radiation segmentectomy followed by liver transplantation demonstrated that a complete radiologic response, based on modified RECIST criteria, was associated with complete pathologic response (> 99% tumor necrosis) [21]. In another single-center histopathologic study of 35 patients with 38 tumors, complete pathologic tumor necrosis was observed in all tumors classified as having a complete response according to the European Association for the Study of the Liver necrosis criteria [22]. The present histopathologic study confirms similar findings in patients with iCCA.

Concurrent systemic therapy has been associated with improved tumor necrosis in the present study, consistent with prior studies suggesting improved clinical outcomes with combined treatment. Based on the multicenter phase II MISPHEC trial, first-line treatment with gemcitabine, cisplatin, and resin-based radioembolization achieved a MST of 22 months and a disease control rate of 98% [11]. A single-center prospective study including 24 patients with iCCA treated with first-line glass microsphere TARE without systemic therapy reported a MST of 19.4 months [13]. In contrast, another single-center study of 13 patients with iCCA treated with first-line gemcitabine, cisplatin, capecitabine, and glass Y90 radioembolization reported an MST of 24 months [10]. Given the tumor biology described above, TARE alone may be insufficient to achieve complete tumor necrosis, particularly in advanced-stage disease. The addition of systemic therapy may provide additive, if not synergistic, effects. Notably, gemcitabine, commonly used as a first-line agent for iCCA, is considered a radiosensitizer [23]. Furthermore, because these tumors are often diagnosed at a late stage, there may be occult extrahepatic disease that is not detectable on cross-sectional imaging. This could help explain the high rate of post-surgical recurrence, even among transplant recipients. The incorporation of systemic therapy may aid in controlling microscopic extrahepatic disease and potentially improve recurrence-free survival.

The results of the present study should be interpreted with several important caveats. First, the study population is heterogeneous, with most patients undergoing multimodal therapy that included both radioembolization and systemic treatments. Multiple treatment deliveries were often required due to the presence of multiple vascular feeders. This reflects the typical presentation of unresectable iCCA, which frequently necessitates a combination of locoregional and systemic approaches. Consequently, identifying a clear dose threshold, such as those reported in prior HCC studies, is challenging, particularly because those studies often included treatment-naïve, solitary lesions managed with TARE alone. Additionally, patients who ultimately underwent resection or transplantation were more likely to have responded to therapy, introducing potential selection bias. Finally, this is a single-center, retrospective study, which may limit the generalizability of the findings.

## Conclusion

Based on tumor explant data, TARE can result in substantial tumor necrosis in the treatment of iCCA, although complete necrosis is uncommon with current techniques. The addition of systemic therapy appears to be associated with a higher likelihood of achieving extensive tumor necrosis. Survival outcomes among CCA patients who received TARE and eventually surgery were promising, although recurrence rate remains high.

## Data Availability

The datasets generated during and/or analysed during the current study are available from the corresponding author on reasonable request.
